# Analysis of the Relationship between Adult Asthma and Stroke: A Longitudinal Follow-Up Study Using the Korean National Sample Cohort

**DOI:** 10.1155/2019/8919230

**Published:** 2019-06-17

**Authors:** So Young Kim, Hyun Lim, Jae-Sung Lim, Hyo Geun Choi

**Affiliations:** ^1^Department of Otorhinolaryngology-Head & Neck Surgery, CHA Bundang Medical Center, CHA University, Seongnam, Republic of Korea; ^2^Department of Internal Medicine, Hallym University College of Medicine, Anyang, Republic of Korea; ^3^Department of Neurology, Hallym University Sacred Heart Hospital, Anyang, Republic of Korea; ^4^Department of Otorhinolaryngology-Head & Neck Surgery, Hallym University College of Medicine, Anyang, Republic of Korea

## Abstract

Several previous studies demonstrated the risk of stroke in asthma patients. The aim of this study was to evaluate the risk of hemorrhagic and ischemic stroke in asthma patients, independent of age, sex, income, region of residence, and past medical histories. The Korean Health Insurance Review and Assessment Service-National Sample Cohort from 2002 through 2013 was used. Overall, 111,364 asthma patients ≥ 20 years old were matched to 111,364 control participants for age, sex, income, region of residence, hypertension, diabetes, and dyslipidemia. Asthma was classified using ICD-10 codes (J45 and J46) and medication history. The admission histories were investigated for hemorrhagic stroke (I60-I62) and ischemic stroke (I63) using ICD-10 codes. The crude and adjusted (age, sex, income, region of residence, hypertension, diabetes, dyslipidemia, ischemic heart disease, and depression) hazard ratios (HRs) for hemorrhagic and ischemic stroke in asthma patients were analyzed using a Cox proportional hazards model. Subgroup analyses were conducted according to age and sex. Hemorrhagic and ischemic stroke were found in 0.7% (795/117,364) and 2.4% (922/117,364) of the asthma group and in 0.8% (922/117,364) and 2.6% (93,079/117,364) of the control group, respectively. The asthma group demonstrated adjusted HRs of 0.86 (95% confidence interval [CI] = 0.78-0.94, p = 0.002) for hemorrhagic stroke and 0.91 (95% CI = 0.86-0.95, p = 0.002) for ischemic stroke. None of the subgroups of asthma patients showed higher HRs for stroke. Asthma did not elevate the risk of either hemorrhagic or ischemic stroke.

## 1. Introduction

Asthma is an airway disorder with a cluster of related symptoms of reversible expiratory airflow limitation and airway hyperresponsiveness. As much as 5-10% of the general population suffers from asthma [1]. In Korea, the prevalence of asthma in the adult population was estimated to be approximately 5.0-5.7% [2]. Allergic airway inflammation is known to cause asthma. However, multiple pathophysiologic mechanisms have been suggested to be associated with asthma. Asthma has heterogeneous phenotypes with correspondingly varying pathophysiologies [1].

Chronic inflammation in asthma patients could influence the pathogenesis of cardiovascular disorders because of their common pathophysiology of inflammation and immune dysfunction [3, 4]. The most common aetiology of coronary heart disease is atherosclerosis, which is induced by a chronic inflammatory condition [3]. In line with this, several studies suggested an elevated risk of cardiovascular diseases, including myocardial infarction, angina, heart failure, and stroke, in asthma [5, 6]. Asthma patients demonstrated a 1.57-fold higher risk of incident cardiovascular disease than the control group (95% confidence interval [CI] = 1.01-2.45, p = 0.045) [6]. Cardiovascular diseases are related to the risk of stroke and share a common pathophysiology with stroke [7]. Therefore, a relationship between stroke and asthma could be postulated.

Several previous studies demonstrated the risk of stroke in asthma patients [8, 9]. A population cohort study using health claim codes indicated a 1.37-fold higher risk of stroke in asthma patients than in the control group (95% CI = 1.27-1.48) [9]. However, they did not consider a number of potential confounding factors, including socioeconomic status and medications. A meta-analysis reported that asthma was related to the pooled hazard ratio (HR) of 1.32 for stroke (95% CI = 1.13-1.54) [8]. However, this meta-analysis study was based on less than 2000 asthma cases if the abovementioned health claim data are excluded. Other studies that suggested the association between asthma and stroke classified the asthma group using a self-reported survey [5, 10]. Thus, it is possible that the relationship between asthma and stroke was overestimated in previous studies. Stroke has multiple etiologies, including embolism, artery occlusion, and other causes, in addition to atherosclerosis and inflammation [11]. In addition, because the subtypes of stroke, such as ischemic and hemorrhagic stroke, have different pathophysiologies, the association of stroke with asthma should be investigated differentially according to the stroke subtype. We hypothesized that the risk of ischemic and hemorrhagic stroke in asthmatic adults could be different from the risk in nonasthmatic adults but might have been overestimated in previous studies.

## 2. Materials and Methods

### 2.1. Study Population and Data Collection

The ethics committee of Hallym University (2017-I102) approved the use of these data. The Institutional Review Board waived the requirement for written informed consent.

This national cohort study used data from the Korean National Health Insurance Service-National Sample Cohort (NHIS-NSC). A detailed description of these data was given in our previous studies [12, 13].

### 2.2. Participant Selection

From 1,125,691 cases with 114,369,638 medical claim codes, we included participants who were diagnosed with asthma (ICD-10: J45) or status asthmaticus (J46) from 2002 through 2013. From these participants, we selected participants who were diagnosed with asthma by a physician more than 2 times and who were treated with asthma-related medications, including inhaled corticosteroids (ICSs), ICSs combined with long-acting *β*2-agonists (LABAs), oral leukotriene antagonists (LTRAs), short-acting *β*2-agonists (SABAs), systemic LABAs, xanthine derivatives, and systemic corticosteroids (n = 230,764). This method has been modified from a previous study [2]. Therefore, the participants were followed up to 12 years.

The history of admission for hemorrhagic stroke (I60: subarachnoid hemorrhage, I61: intracerebral hemorrhage, and I62: other nontraumatic intracranial hemorrhage) and ischemic stroke (I63: cerebral infarction) was identified using ICD-10 codes. We selected the participants who were treated ≥ 1 times. These methods were used in other studies that evaluated the incidence of stroke in Korea [14, 15].

**Figure 1:**
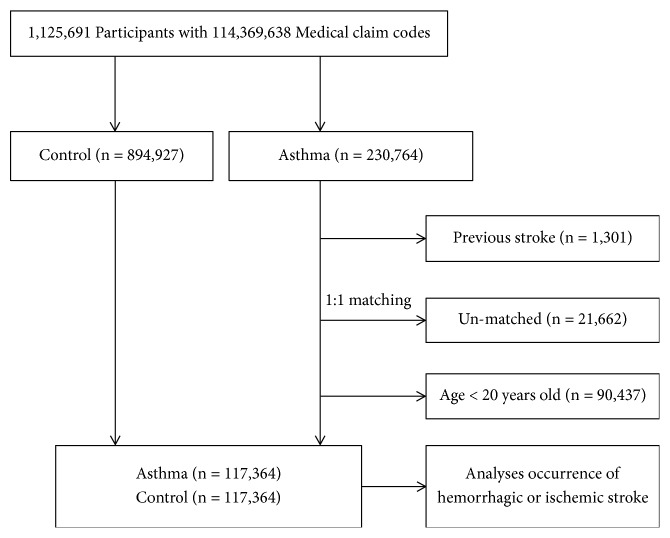
A schematic illustration of the participant selection process that was used in the present study. Out of a total of 1,125,691 participants, 117,364 of asthma participants were matched with 117,364 control participants by age, group, sex, income group, region of residence, and past medical histories.

The asthma participants were matched 1:1 with the participants (control group) who had never been diagnosed with asthma from 2002 through 2013 among this cohort. The control groups were selected from the mother population (n = 894,927). The matches were processed for age, group, sex, income group, region of residence, and past medical histories (hypertension, diabetes, and dyslipidemia). To prevent selection bias when selecting the matched participants, the control group participants were sorted using a random number order, and they were then selected from top to bottom. It was assumed that the matched control participants were involved at the same time as each matched asthma participant (index date). Therefore, the control group participants who died before the index date were excluded. In both the asthma and control groups, the participants who had histories of hemorrhagic or ischemic stroke before the index date were excluded. In the asthma group, 1,301 participants were excluded. The asthma participants for whom we could not identify enough matching participants were excluded (n = 21,662). We excluded participants under 20 years old (n = 90,437). Finally, 1:1 matching resulted in the inclusion of 117,364 asthma participants and 117,364 control participants (Figure 1). However, the participants were not matched for the ischemic heart disease and depression histories because strict matching increased the number of dropouts caused by a lack of matched control participants.

### 2.3. Variables

The age groups were classified using 5-year intervals: 20-24, 25-29, 30-34…, and 85+ years of age. A total of 14 age groups were designated. The income groups were initially divided into 41 classes (one health aid class, 20 self-employment health insurance classes, and 20 employment health insurance classes). These groups were recategorized into 5 classes (classes 1 [lowest income]-5 [highest income]). Region of residence was divided into 16 areas according to administrative district. These regions were regrouped into urban (Seoul, Busan, Daegu, Incheon, Gwangju, Daejeon, and Ulsan) and rural (Gyeonggi, Gangwon, Chungcheongbuk, Chungcheongnam, Jeollabuk, Jeollanam, Gyeongsangbuk, Gyeongsangnam, and Jeju) areas.

The past medical histories of participants were evaluated using ICD-10 codes. For the accuracy of diagnosis, hypertension (I10 and I15), diabetes (E10-E14), and dyslipidemia (E78) were considered for participants who were treated ≥ 2 times. Ischemic heart disease (I24 and I25) was considered for participants who were treated ≥ 1 time. Depression was defined by the use of the ICD-10 codes F31 (bipolar affective disorder) through F39 (unspecified mood disorder) by a psychiatrist ≥ 2 times.

### 2.4. Statistical Analyses

A chi-square test was used to compare the rate of general characteristics between the asthma and control groups.

To analyze the HR for hemorrhagic stroke and ischemic stroke in patients with asthma, a Cox proportional hazards model was used. In these analyses, crude (simple) and adjusted (age, sex, income, region of residence, hypertension, diabetes, dyslipidemia, ischemic heart disease, and depression) models were used. The 95% CI was calculated.

For the subgroup analyses, we divided the participants by age and sex (20-39 years old, 40-59 years old, and 60+ years old; men and women) and according to the medication histories (systemic corticosteroid + inhaled corticosteroid [ICS] [with/without other medication], systemic corticosteroid [with/without other medication], ICS [with/without other medication], other medications only).

Two-tailed analyses were conducted, and p-values less than 0.05 were considered to indicate significance. The results were statistically analyzed using SPSS v. 21.0 (IBM, Armonk, NY, USA).

## 3. Results

The rate of hemorrhagic stroke was lower in the asthma group (0.7% [795/117,364]) than in the control group (0.8% [922/117,364], p = 0.002, Table 1). The rate of ischemic stroke was also lower in the asthma group (2.4% [2,849/117,364]) than in the control group (2.6% [3,079/117,364], p = 0.002). The general characteristics (age, sex, income, region of residence, and histories of hypertension, diabetes, and dyslipidemia) of participants were identical due to matching (p = 1.000). Higher rates of histories of ischemic heart disease and depression were observed in the asthma group (all p values < 0.05).

The adjusted HR for hemorrhagic stroke in the asthma group was 0.86 (95% CI = 0.78-0.94, p = 0.002), and the adjusted HR for ischemic stroke in the asthma group was 0.91 (95% CI = 0.86-0.95, p = 0.002, Table 2).

In all subgroup analyses according to age and sex, the crude and adjusted HRs of haemorrhagic or ischaemic stroke were not higher in the asthma group (Table 3). The middle-aged men subgroup showed a lower adjusted HR than the control group (adjusted HR = 0.76, 95% CI = 0.64 – 0.91, p = 0.002). Other age and sex subgroups did not demonstrate significant HRs of asthma for stroke.

According to the medication histories, the asthma patients with systemic corticosteroid use without inhaled corticosteroid, with/without other medication demonstrated 0.75 (95% CI = 0.76 – 0.88) and 0.79 (0.73 – 0.85) adjusted HRs for hemorrhagic and ischemic stroke, respectively (Table 4).

## 4. Discussion

Asthma did not increase the risk of either ischemic or hemorrhagic stroke in the ≥ 20-year-old population in the present study, contrary to our expectations. The results were consistent in the age and sex subgroups. These results contradict previous studies. Because both asthma and stroke are influenced by numerous comorbidities, including hypertension, diabetes, and dyslipidemia, the matching of these comorbidities could minimize the confounding effects of these confounders and result in the lack of a significant relationship between asthma and stroke found in this study. This matching of the control group for past medical histories improved the previous findings. In addition, the socioeconomic status was matched between the asthma and control groups in the present study. Because the NHIS data are based on hospital visits, medical accessibility, which is largely determined by socioeconomic status, must be comparable between asthma and control groups to prevent selection bias.

The relationship between adult-onset asthma and other cardiovascular diseases, such as coronary heart disease, could be attributed to the elevated risk of stroke in asthmatic adults in previous studies. When considering several cardiovascular diseases concurrently in a previous study, only coronary heart disease was related to adult-onset asthma (odds ratio (OR) = 2.26, 95% CI = 1.2 -4.2) [16]. The possible confounding effects of cardiovascular risk factors could mediate the link between asthma and stroke in a previous study. Past medical histories of hypertension, diabetes, and hyperlipidemia are risk factors for stroke that need to be matched between the control and asthma groups [17]. Although a previous study adjusted for past medical histories of hypertension, diabetes, and other potential confounders and cardiovascular risk factors, the confounding effects might stem from the different characteristics between the unmatched control and asthma groups [9, 18]. In addition, subclinical heart diseases could initially manifest as asthma symptoms. Cardiac asthma is an asthma symptom of wheezing and coughing due to pulmonary edema and pulmonary vascular congestion in congestive heart failure patients [19, 20]. Because the present study considered asthma that was diagnosed by a physician and medication histories, the misclassification of asthma might be minimized.

The association between asthma and stroke has been controversial in previous studies. In contrast to the present results, some prior studies reported an elevated risk of stroke in asthma patients [5, 8–10, 18]. In a meta-analysis, the asthma patients showed a higher risk of stroke than the control group [8]. However, the risk of stroke in asthma patients varied among studies (I^2^=80.4%) [8]. The heterogeneous results among studies could be explained by the differences in the characteristics of the study population and the definitions for asthma and strokes. A few studies classified asthma using a self-reported survey [5, 10]. Two nationwide population cohort studies using the National Health Insurance Research Database and health check-up program used medical claim codes, which were diagnosed by specialists [9, 18]. However, the studies neither matched nor adjusted for socioeconomic factors.

Similar to this study, a few prior studies reported limited or no association of asthma with stroke [6, 16]. A cross-sectional study demonstrated no significant association between asthma and stroke [16]. No significant relationship was observed between adult-onset asthma and stroke in that study [16]. The researchers reported that adult-onset asthma, but not child-onset asthma, was associated with overall cardiovascular diseases (OR = 2.07, 95% CI = 1.2-3.7 for adult-onset asthma, OR = 1.00, 95% CI = 0.28-3.60 for child-onset asthma) [16]. Another previous study demonstrated that the relationship between cardiovascular diseases and asthma was limited to adult-onset asthma and did not exist for early-onset asthma. A large cohort study reported that the late-onset subtype of asthma but not early-onset asthma was associated with an increased risk of cardiovascular disease (HR = 1.57, 95% CI = 1.01-2.45 p = 0.045 for late-onset asthma, OR = 0.94, 95% CI = 0.46-1.92, p = 0.83 for early-onset asthma) [6].

The relationship of asthma with cardiovascular diseases, including stroke, could be restricted to some endotypes of asthma, such as adult-onset asthma. Because this study did not discriminate between early- and adult-onset asthma, the effects of asthma on stroke could be limited compared to those in previous studies. Because the pathophysiologic causes of asthma are different according to the endotype of asthma, the association of stroke with asthma could not be generalized to all endotypes of asthma. Th2-associated or early-onset allergic Th2 asthma is known to be induced by allergic and intrinsic factors [21]. Therefore, early-onset asthma might have little influence on the risk of stroke. However, other phenotypes of asthma, such as non-Th2, neutrophilic, and obesity-related asthma, are associated with extrinsic factors and systemic inflammatory diseases [1, 22]. In adult-onset asthma, the systemic inflammatory response and irritant factors could affect the risk of stroke.

In a subgroup analysis according to medication history, the asthma patients with systemic corticosteroid use without inhaled corticosteroid, with/without other medication, showed lower risks of both hemorrhagic and ischemic stroke than the control groups in this study. This result could be partially due to the protective effects of asthma medication. Previous studies suggested the protective effect of asthma treatment, particularly ICS, on atherosclerosis, probably due to the anti-inflammatory effects on the vascular walls [23, 24]. Carotid intima-media thickness and the prevalence of carotid plaque were lower in asthma patients [23]. In particular, asthma patients with ICS showed reduced risk of carotid atherosclerosis [23]. Furthermore, ICS use had protective roles for cardiovascular and all-cause mortality in women with asthma [25]. In this study, the high number of asthma patients with systemic corticosteroid use without inhaled corticosteroid (n = 143,778) might potentiate the statistical power. In addition, because the subgroup analysis on medication history was focused on the use of steroid, the study population was large in this subgroup. These subgroup patients could also use other asthma medications. Moreover, asthma patients with systemic steroid might have moderate to severe symptoms of asthma. Thus, steps towards establishing a healthy life style, such as cessations of smoking and alcohol, could have protective effects for stroke in these asthma patients.

According to age and sex, most subgroups did not show significant difference on the risk of hemorrhagic or ischemic stroke in this study. Only the middle-aged men subgroup demonstrated lower HR of asthma for ischemic stroke than the control group. Although this study was based on a large study population, it was possible that the small effect size, which was shown in the total population analysis, might have lost statistical significance in the subgroup analysis.

The large study population potentiated the statistical power in the present study. Moreover, nationwide, representative populations were used in this study. Because the NHIS system is managed by the Korean government and legally registers all medical records of all Korean citizens, no missing participants were anticipated. A statistician confirmed that the sample was a representative population of HIRA-NSC, and its validity was published in a previous study [26]. In addition to the large, representative cohort study, this study used an appropriate control group matched for age, sex, income, region of residence, and past medical histories of hypertension, diabetes, and dyslipidemia. Although adjustment of past medical history might attenuate the possible confounding effects, there could be confounding effects of these factors if these past medical histories are not matched [27]. The socioeconomic factors of income and region of residence should be matched between the control and asthma groups because these factors determine medical accessibility. The objective disease classifications were another virtue of the present study. Asthma and stroke were defined using ICD-10 codes and medication history in this study. Both asthma and stroke classifications were verified in previous studies [2, 14, 15].

However, a few limitations should be considered when interpreting the present results. For asthma, data on the phenotypes of asthma (Th2 low and Th2 high) and asthma severity were not available in the present study. This heterogeneity in the disease groups was also retained in the stroke groups. The brain lesion related to stroke could not be classified. In addition, the severity of stroke and management histories could not be assessed in this study. However, this study differentiated ischemic stroke and hemorrhagic stroke. Lastly, lifestyle factors, such as body mass index, smoking, and alcohol consumption, were not considered in the present study.

## 5. Conclusion

Neither hemorrhagic nor ischemic stroke was increased in asthma patients compared to their incidence in control subjects matched for age, sex, income, region of residence, and past medical histories. The results were consistent in the age and sex subgroups.

## Figures and Tables

**Table 1:** General characteristics of the participants.

Characteristics	Total participants		
	Asthma (n, %)	Control (n, %)	p-value
Age (years old)			1.000
20-24	4,568 (3.9)	4,568 (3.9)	
25-29	7,346 (6.3)	7,346 (6.3)	
30-34	10,959 (9.3)	10,959 (9.3)	
35-39	11,159 (9.5)	11,159 (9.5)	
40-44	10,923 (9.3)	10,923 (9.3)	
45-49	11,463 (9.8)	11,463 (9.8)	
50-54	11,254 (9.6)	11,254 (9.6)	
55-59	10,743 (9.2)	10,743 (9.2)	
60-64	10,701 (9.1)	10,701 (9.1)	
65-69	10,606 (9.0)	10,606 (9.0)	
70-74	8,371 (7.1)	8,371 (7.1)	
75-79	5,219 (4.4)	5,219 (4.4)	
80-84	2,796 (2.4)	2,796 (2.4)	
85+	1,256 (1.1)	1,256 (1.1)	
Sex			1.000
Male	42,644 (36.3)	42,644 (36.3)	
Female	74,720 (63.7)	74,720 (63.7)	
Income			1.000
1 (lowest)	19,463 (16.6)	19,463 (16.6)	
2	17,485 (14.9)	17,485 (14.9)	
3	20,976 (17.9)	20,976 (17.9)	
4	26,717 (22.8)	26,717 (22.8)	
5 (highest)	32,723 (27.9)	32,723 (27.9)	
Region of residence			1.000
Urban	53,742 (45.8)	53,742 (45.8)	
Rural	63,622 (54.2)	63,622 (54.2)	
Hypertension	45,542 (38.8)	45,542 (38.8)	1.000
Diabetes	23,449 (20.0)	23,449 (20.0)	1.000
Dyslipidemia	33,128 (28.2)	33,128 (28.2)	1.000
Ischemic heart disease	8,920 (7.6)	6,971 (5.9)	<0.001*∗*
Depression	14,188 (12.1)	10,175 (8.7)	<0.001*∗*
Hemorrhagic stroke	795 (0.7)	922 (0.8)	0.002*∗*
Ischemic stroke	2,849 (2.4)	3,079 (2.6)	0.002*∗*

*∗*Chi-square test or Fisher's exact test. Significance at p < 0.05.

**Table 2:** Crude and adjusted hazard ratios (95% confidence interval) of asthma for hemorrhagic and ischemic stroke.

Characteristics	Hemorrhagic stroke				Ischemic stroke			
	Crude	p-value	Adjusted†	p-value	Crude	p-value	Adjusted†	p-value
Asthma	0.86 (0.78-0.95)	0.002*∗*	0.86 (0.78-0.94)	0.002*∗*	0.92 (0.88-0.97)	0.002*∗*	0.91 (0.86-0.95)	0.002*∗*
Control	1.00		1.00		1.00		1.00	

*∗* Cox proportional hazards regression model, significance at p < 0.05.

† Adjusted model for age, sex, income, region of residence, hypertension, diabetes, hyperlipidemia, ischemic heart disease, and depression histories.

Table 2 is reproduced from Choi HG et al. (2018), (doi: 10.1016/j.maturitas.2018.08.008. Epub 2018 Aug 23.).

**Table 3:** Subgroup analysis of crude and adjusted hazard ratios (95% confidence interval) of asthma for hemorrhagic and ischemic stroke according to age and sex.

Characteristics	Hemorrhagic stroke				Ischemic stroke			
	Crude	p-value	Adjusted†	p-value	Crude	p-value	Adjusted†	p-value
*Young men (20-39 years old, n = 22,396)*								
Asthma	0.90 (0.47-1.72)	0.739	0.88 (0.45-1.69)	0.689	0.70 (0.35-1.39)	0.305	0.69 (0.34-1.37)	0.282
Control	1.00		1.00		1.00		1.00	
*Young women (20-39 years old, n = 45,668)*								
Asthma	1.10 (0.61-1.98)	0.763	1.04 (0.58-1.89)	0.886	1.20 (0.66-2.17)	0.547	1.16 (0.64-2.10)	0.623
Control	1.00		1.00		1.00		1.00	
*Middle-aged men (40-59 years old, n = 32,316)*								
Asthma	0.96 (0.72-1.27)	0.774	0.94 (0.71-1.25)	0.674	0.79 (0.67-0.94)	0.006*∗*	0.76 (0.64-0.91)	0.002*∗*
Control	1.00		1.00		1.00		1.00	
*Middle-aged women (40-59 years old, n = 56,450)*								
Asthma	1.12 (0.89-1.42)	0.337	1.11 (0.88-1.40)	0.387	1.01 (0.85-1.21)	0.899	0.98 (0.82-1.17)	0.842
Control	1.00		1.00		1.00		1.00	
*Old men (60+ years old, n = 30,576)*								
Asthma	0.74 (0.62-0.90)	0.002*∗*	0.74 (0.62-0.89)	0.002*∗*	0.90 (0.82-0.98)	0.016*∗*	0.89 (0.81-0.97)	0.008*∗*
Control	1.00		1.00		1.00		1.00	
*Old women (60+ years old, n = 47,322)*								
Asthma	0.81 (0.70-0.94)	0.006*∗*	0.82 (0.70-0.95)	0.008*∗*	0.95 (0.88-1.02)	0.158	0.94 (0.87-1.01)	0.080
Control	1.00		1.00		1.00		1.00	

*∗* Cox proportional hazards regression model, significance at p < 0.05.

† Adjusted model for age, sex, income, region of residence, hypertension, diabetes, hyperlipidemia, ischemic heart disease, and depression histories.

**Table 4:** Subgroup analysis of crude and adjusted hazard ratios (95% confidence interval) of asthma for hemorrhagic and ischemic stroke according to medication histories.

Characteristics	Hemorrhagic stroke				Ischemic stroke			
	Crude	p-value	Adjusted†	p-value	Crude	p-value	Adjusted†	p-value
*Systemic corticosteroid and inhaled corticosteroid use with/without other medication (n = 81,556)*								
Asthma	1.00 (0.87-1.14)	0.983	1.01 (0.88-1.15)	0.946	1.03 (0.96-1.11)	0.391	1.01 (0.94-1.09)	0.742
Control	1.00		1.00		1.00		1.00	
*Systemic corticosteroid use without inhaled corticosteroid, with/without other medication (n = 143,778)*								
Asthma	0.76 (0.66-0.87)	<0.001*∗*	0.75 (0.65-0.86)	<0.001*∗*	0.81 (0.76-0.88)	<0.001*∗*	0.79 (0.73-0.85)	<0.001*∗*
Control	1.00		1.00		1.00		1.00	
*Inhaled corticosteroid use without systemic corticosteroid, with/without other medication (n = 5,658)*								
Asthma	0.75 (0.34-1.63)	0.468	0.79 (0.36-1.72)	0.555	0.82 (0.55-1.23)	0.344	0.85 (0.56-1.27)	0.424
Control	1.00		1.00		1.00		1.00	
*Other medications without systemic corticosteroid and inhaled corticosteroid (n = 3,736)*								
Asthma	0.08 (0.01-0.65)	0.017*∗*	0.08 (0.01-0.64)	0.017*∗*	1.05 (0.64-1.72)	0.852	1.15 (0.70-1.89)	0.587
Control	1.00		1.00		1.00		1.00	

*∗* Cox proportional hazards regression model, significance at p < 0.05.

† Adjusted model for age, sex, income, region of residence, hypertension, diabetes, hyperlipidemia, ischemic heart disease, and depression histories.

## Data Availability

The raw data of experiments used to support the findings of this study are available from the corresponding author upon request.
